# Distinct Binding Properties of Neutravidin and Streptavidin Proteins to Biotinylated Supported Lipid Bilayers: Implications for Sensor Functionalization

**DOI:** 10.3390/s22145185

**Published:** 2022-07-11

**Authors:** Tun Naw Sut, Hyeonjin Park, Dong Jun Koo, Bo Kyeong Yoon, Joshua A. Jackman

**Affiliations:** 1 School of Chemical Engineering and Translational Nanobioscience Research Center, Sungkyunkwan University, Suwon 16419, Korea; suttunnaw@skku.edu (T.N.S.); guswls887@skku.edu (H.P.); dongjkoo@skku.edu (D.J.K.); 2 School of Healthcare and Biomedical Engineering, Chonnam National University, Yeosu 59626, Korea

**Keywords:** supported lipid bilayer, neutravidin, streptavidin, protein adsorption, surface functionalization

## Abstract

The exceptional strength and stability of noncovalent avidin-biotin binding is widely utilized as an effective bioconjugation strategy in various biosensing applications, and neutravidin and streptavidin proteins are two commonly used avidin analogues. It is often regarded that the biotin-binding abilities of neutravidin and streptavidin are similar, and hence their use is interchangeable; however, a deeper examination of how these two proteins attach to sensor surfaces is needed to develop reliable surface functionalization options. Herein, we conducted quartz crystal microbalance-dissipation (QCM-D) biosensing experiments to investigate neutravidin and streptavidin binding to biotinylated supported lipid bilayers (SLBs) in different pH conditions. While streptavidin binding to biotinylated lipid receptors was stable and robust across the tested pH conditions, neutravidin binding strongly depended on the solution pH and was greater with increasingly acidic pH conditions. These findings led us to propose a two-step mechanistic model, whereby streptavidin and neutravidin binding to biotinylated sensing interfaces first involves nonspecific protein adsorption that is mainly influenced by electrostatic interactions, followed by structural rearrangement of adsorbed proteins to specifically bind to biotin functional groups. Practically, our findings demonstrate that streptavidin is preferable to neutravidin for constructing SLB-based sensing platforms and can improve sensing performance for detecting antibody–antigen interactions.

## 1. Introduction

**Figure 1:**
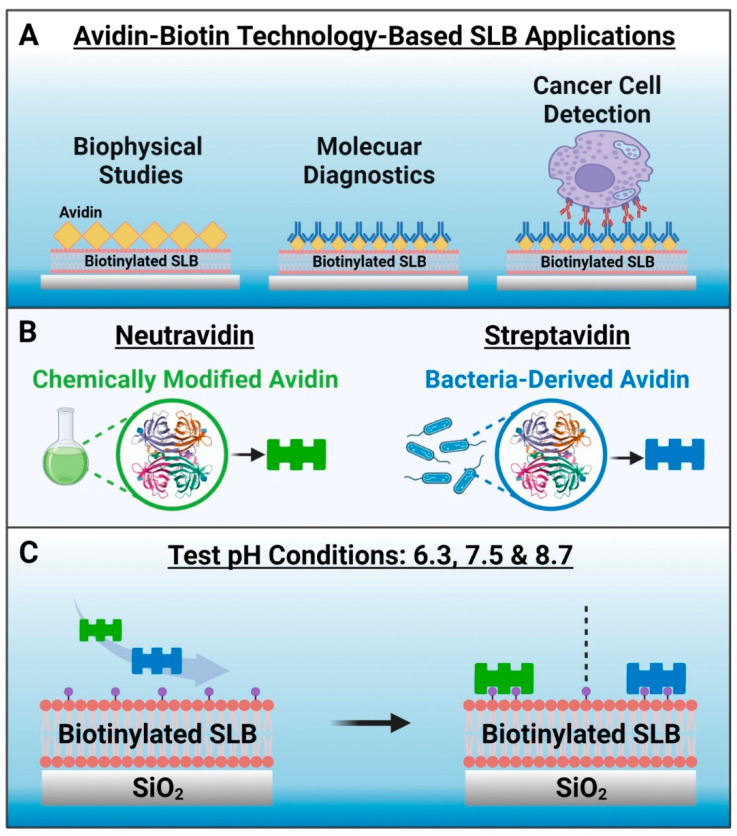
Conceptual overview and rationale to investigate neutravidin and streptavidin adsorption onto biotinylated supported lipid bilayer (SLB) biosensing platforms. (**A**) Examples of avidin–biotin bioconjugation strategies used in SLB-based applications. (**B**) Schematic illustrations of methods to produce neutravidin and streptavidin, which are avidin analogues. (**C**) Experimental strategy. The tested pH conditions were 6.3, 7.5 and 8.7, and the biotinylated SLB was formed on a silica surface and contained 5 mol% biotinylated lipids. Note that no Neu or Strep binding occurs to the SLB in the absence of biotinylated lipid.

Avidin–biotin binding is an exceptionally strong and naturally occurring noncovalent interaction that has been utilized in numerous biosensing and biotechnology applications [1,2,3,4,5,6]. The highly specific and stable nature of avidin–biotin binding across various environments (e.g., diverse temperature and pH ranges) enables the specific capture and recognition of biomolecules that can be useful for applications such as biosensing, immunoassays, and targeted drug delivery. Moreover, the avidin–biotin interaction has been utilized in nanoscale drug delivery, diagnostic, and biosensor systems due to its favorable merits such as ease of functionalization, efficiency, and stability [7,8,9,10,11]. The main feature of these nanotechnology-focused applications lies in the modification of interacting surfaces with biotin- and/or avidin-conjugated biomolecules, either directly or through multi-step binding sequences and the specifics of the surface modification can be tuned according to the application needs. One emerging use of avidin–biotin technology is in the field of supported lipid bilayer (SLB) coatings [12], which are antifouling cell membrane mimics that are formed through the self-assembly of phospholipid molecules on solid surfaces and are widely used in biosensor development [13,14,15,16]. Generally, biotinylated lipids are incorporated in the SLB that acts as a platform upon which avidin or related protein analogues are specifically attached via biotin coupling, and such platforms can be used in various biosensing applications ranging from fundamental biophysical studies to molecular diagnostics and cancer cell detection [17,18,19,20,21,22,23,24,25,26,27,28] (Figure 1A).

Avidin is an egg-white glycoprotein that has four binding sites for biotin along with a high isoelectric point (pI) around pH 10.5 (ref. [29]). Although avidin has strong biotin binding affinity, the high pI of avidin makes it prone to nonspecific adsorption of negatively charged molecules in physiological pH conditions. Therefore, non-glycosylated analogues of avidin with lower pI values such as neutravidin (Neu) [30] and streptavidin (Strep) [31] that are derived from chemical modification of avidin and from bacteria, respectively, have been engineered and are often used instead of avidin (Figure 1B). For example, it has been reported that Neu-based nanocomplexes are more efficient for nucleic acid delivery than avidin-based nanocomplexes [8]. Since their biotin binding affinity in solution is similar [8], Neu and Strep are often used interchangeably for biosensing applications, including SLB surface functionalization. In the latter respect, it is noteworthy that Nguyen et al. have reported that Strep has a more than 1.6 times greater intrinsic binding affinity to a biotinylated SLB, as compared to that of Neu, according to second-harmonic generation spectroscopic measurements [12]. Such findings motivate the need to evaluate the corresponding adsorption kinetics of specific Neu and Strep binding to biotinylated SLBs, not only for fundamental understanding but to also identify suitable strategies for robust surface functionalization in biosensing application contexts.

Herein, we conducted quartz crystal microbalance-dissipation (QCM-D) experiments to investigate Neu and Strep binding to biotinylated SLBs in different pH conditions. Since the two proteins have different pI values and electrostatic forces can have a strong influence on protein adsorption in general [32], we decided to test three pH conditions of 6.3, 7.5, and 8.7, as discussed below, along with a total of three proteins: two commercially available versions of Strep and the one commercially available version of Neu. For biotinylated SLB fabrication, we used silica as the sensor surface and a biotinylated lipid fraction of 5 mol%, which is around the optimal biotin fraction for SLB-based biosensing platforms [33,34]. Figure 1C outlines the study design, and we used the bicelle method [35,36] to fabricate biotinylated SLBs along with the QCM-D technique [37] to characterize all experimental stages. This measurement approach led us to identify the robustness and reliability of Strep adsorption under all tested pH conditions, while Neu showed more variable pH-dependent adsorption behavior.

## 2. Materials and Methods

*Reagents.* Neutravidin (referred to as Neu, Catalog No.: 31000) was obtained from Thermo Fisher Scientific (Waltham, MA, USA). The first streptavidin (referred to as Strep 1, Catalog No.: S203) was obtained from Leinco Technologies (St. Louis, MO, USA), and the second streptavidin (referred to as Strep 2, Catalog No.: PRO-791) was obtained from ProSpec-Tany TechnoGene Ltd. (Ness-Ziona, Israel). 1,2-Dioleoyl-*sn*-glycero-3-phosphocholine (DOPC), 1,2-dihexanoyl-*sn*-glycero-3-phosphocholine (DHPC), and 1,2-dioleoyl-*sn*-glycero-3-phosphoethanolamine-N-(biotinyl) (sodium salt) (DOPE-Biotin) lipids in chloroform were obtained from Avanti Polar Lipids (Alabaster, AL, USA). The Tris buffer used in all experiments contained 10 mM Tris and 150 mM NaCl in Milli-Q-treated water (MilliporeSigma, Burlington, MA, USA), and the solution pH was adjusted to 6.3, 7.5, or 8.7, depending on the experiment.

*Sample Preparation.* Bicelles were prepared by the freeze–thaw-vortex cycling method following the hydration of lipids in Tris buffer (pH 7.5), as previously described [36]. The stock bicelles had a q-ratio (molar ratio of long-chain to short-chain phospholipids) of 0.25, i.e., 1 mM of DOPC (including 5 mol% DOPE-Biotin) to 4 mM of DHPC. After bicelle preparation, the sample was diluted 31-times in Tris buffer (pH 7.5) immediately before use for SLB fabrication. Stock solutions of Neu, Strep 1, and Strep 2 were prepared in Milli-Q-treated water and diluted to 1 µM concentration in the Tris buffer with appropriate solution pH immediately before experiment.

*Quartz Crystal Microbalance-Dissipation (QCM-D).* The SLB fabrication and subsequent adsorption processes were tracked by the QCM-D technique and a Q-Sense E4 instrument (Biolin Scientific AB, Gothenburg, Sweden) was used for all experiments, as previously described [37]. Before the experiments, the silica-coated QCM-D sensor chips were prepared as follows: rinsing with water and ethanol, drying with nitrogen gas, and surface treatment for 1 min with oxygen plasma in a vacuum chamber (CUTE-1MPR machine, Femto Science Inc., Hwaseong, Republic of Korea). During the experiments, the temperature of the QCM-D measurement chambers was maintained at 25 °C. The samples were flowed through the measurement chambers by a peristaltic pump (Reglo Digital MS-4/6, Ismatec, Glattbrugg, Switzerland) at a volumetric flow rate of 50 µL/min. The data were collected at odd overtones (3rd to 13th), and the normalized data at the 7th overtone are presented. For data processing, the Q-Tools (Biolin Scientific AB) and OriginPro (OriginLab, Northampton, MA, USA) software programs were used.

## 3. Results and Discussion

### 3.1. Experimental Strategy

We conducted QCM-D experiments to investigate the effects of solution pH on Neu and Strep adsorption onto biotinylated SLBs. We selected pH 7.5 as one of the test pH conditions because it is around physiological pH while the other test pH conditions were selected based on the pI of each protein. We hypothesized that protein adsorption at solution pH conditions around the corresponding pI would involve less electrostatic repulsion compared to higher solution pH conditions above the pI. Within this context, it has been reported that Neu and Strep proteins have pI values of 6.3 (ref. [38]) and around 7 (ref. [39]), respectively, so we selected pH 6.3 as the second test pH condition. Since pH 7.5 is 1.2 pH units higher than the pI of Neu, we also selected pH 8.7 as a third test pH condition for comparison. While Neu is only available from one commercial source, Strep is available from multiple commercial sources, and we selected Strep 1 and 2 as two options that were produced from *Streptomyces avidinii* and *Escherichia coli* bacteria, respectively.

**Figure 2:**
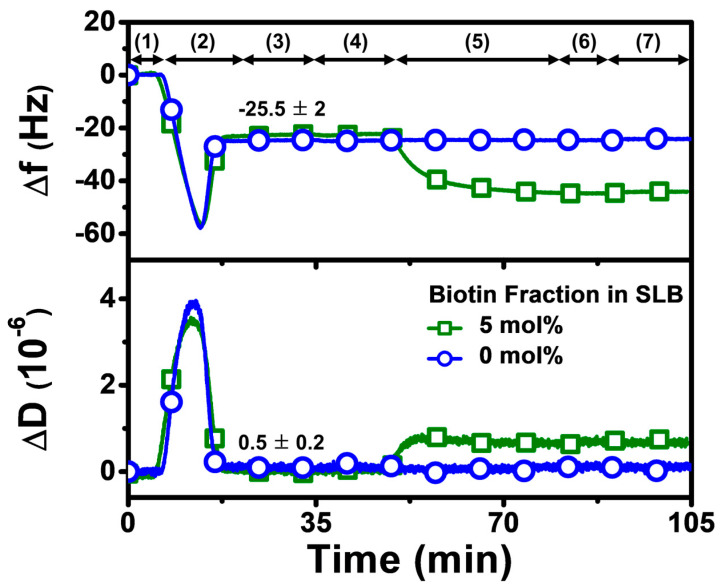
QCM-D tracking of biotinylated SLB fabrication on silica-coated sensor chip surface. Time-resolved QCM-D frequency (Δf) and energy dissipation (ΔD) signals for SLB formation. The initial baseline stage denoted as (1) corresponds to pH 7.5 buffer solution, followed by (2) the addition of lipid bicelles that contain 0 or 5 mol% biotinylated lipid and (3) washing with pH 7.5 buffer. After SLB fabrication, a (4) buffer exchange step was performed to switch to the appropriate test pH, and then (5) 1 µM protein (neutravidin or streptavidin) was added in the test pH condition, and (6) buffer washing with the test pH was performed, before finally (7) rinsing with pH 7.5 buffer in the pH 6.3 and pH 8.7 cases. At stage (3), the listed values correspond to Δf and ΔD shift values for biotinylated SLB fabrication and are reported as mean ± standard deviation from all experiments in the study. The control experiment with the SLB containing 0 mol% biotinylated lipid verified that biotin-binding protein was specific to biotinylated SLBs, and stable adsorption did not occur due to nonspecific interactions alone.

Our overall experimental strategy aimed first to fabricate a biotinylated SLB that contained 5 mol% biotinylated lipid at pH 7.5, before exchanging the solution pH to the appropriate test condition and then adding Neu or Strep protein (Figure 2). Accordingly, the QCM-D experimental protocol had the following steps: (1) baseline established in Tris buffer (pH 7.5); (2) bicelle addition in Tris buffer (pH 7.5); (3) rinsing with Tris buffer (pH 7.5); (4) exchange to appropriate Tris buffer (pH 6.3 or pH 8.7) in applicable cases; (5) 1 µM protein addition in appropriate Tris buffer (pH 6.3, 7.5, or 8.7); (6) rinsing with appropriate Tris buffer (pH 6.3, 7.5, or 8.7); and (7) rinsing with Tris buffer (pH 7.5) in applicable cases. Step 5, involving protein addition, was fixed at 30 min for all tested proteins to compare the protein uptake and corresponding adsorption kinetics, while also identifying suitable conditions for rapid protein attachment.

### 3.2. Adsorption Experiments

The QCM-D technique monitors time-resolved resonance frequency (Δf) and energy dissipation (ΔD) signals of an oscillating, silica-coated quartz crystal sensor chip to track changes in the corresponding mass and viscoelastic properties of the adlayer, respectively [37]. It has been widely used for characterizing SLB formation and is particularly well-suited for characterizing protein attachment to SLBs in a label-free format, since these two measurement signals allow time-dependent kinetic analysis individually. It is also possible to analyze time-independent plots of the two signals, which can lead to insights into adsorbate configuration and conformational changes from a comparative perspective [40]. Since the QCM-D technique is label-free, there is no requirement for protein labeling and comparable measurement response trends for protein attachment have also been obtained with other biosensing techniques such as localized surface plasmon resonance (LSPR) [41], thus establishing the QCM-D as a useful tool for biomacromolecular characterization in the present study. As mentioned above, we first fabricated a biotinylated SLB on the sensor chip surface in Tris buffer (pH 7.5), which resulted in final Δf and ΔD shifts of around −25 ± 3 Hz and less than 1 × 10^−6^, respectively. These values are consistent with typical QCM-D responses for successful SLB formation that are reported in the literature [37]. Afterwards, the solution pH was adjusted accordingly, and then Neu or Strep protein was added, and the corresponding protein adsorption kinetics were scrutinized. Since our focus was to investigate Neu or Strep attachment, we normalized the SLB shifts to zero values at the initial time point in the rest of Section 3.2 and in Section 3.3 to only present the net shifts due to protein adsorption onto the SLB.

#### 3.2.1. pH 7.5

**Figure 3:**
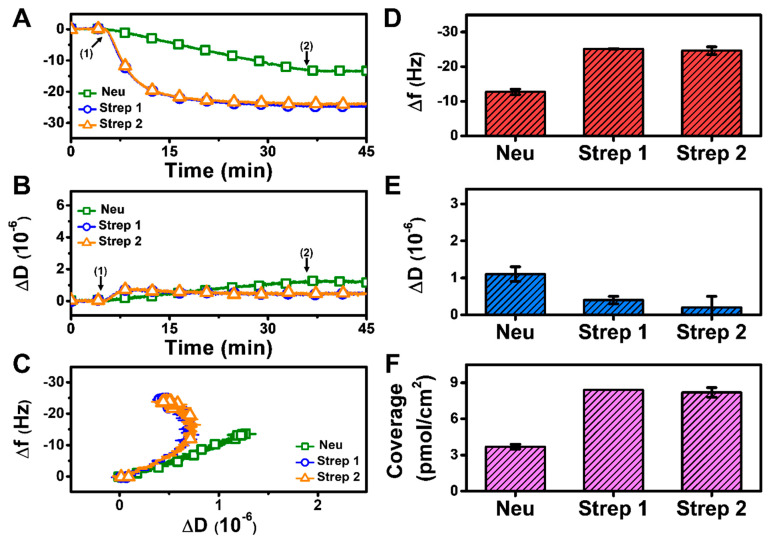
QCM-D measurement responses for Neu and Strep attachment to biotinylated SLB platform at pH 7.5. Time-resolved QCM-D (**A**) frequency (Δf) and (**B**) energy dissipation (ΔD) for protein addition. The initial baselines correspond to the shifts for a biotinylated SLB and protein was added starting at *t* = 5 min (arrow 1), followed by buffer washing from *t* = 35 min (arrow 2). (**C**) Time-independent curves of Δf vs. ΔD shifts corresponding to data in panels (**A**,**B**). Final (**D**) Δf and (**E**) ΔD shift values along with (**F**) maximum surface coverage of adsorbed protein molecules on the biotinylated SLB surface are reported as mean ± standard deviation from *n* = 3 experiments.

At pH 7.5, monotonic adsorption kinetics was observed for both Neu and Strep, with decreasing Δf and increasing ΔD shifts that indicated protein adsorption onto the biotinylated SLB (Figure 3A,B). The Strep adsorption kinetics were also appreciably quicker and reached stable values, whereas Neu adsorption occurred more slowly and did not stabilize within 30 min. The time-independent curves of Δf vs. ΔD (f-D curves) indicate that the Strep adlayers are more rigid than the Neu adlayer since the slopes of the f-D curves for the Strep case are greater than that of the Neu case, i.e., the energy dissipation shift per resonance frequency shift (related to adsorbed protein mass) is smaller (Figure 3C). The final Δf and ΔD shifts for Neu adsorption were around −12.7 ± 0.8 Hz and 1.1 ± 0.2 × 10^−6^, respectively (Figure 3D,E). By contrast, the final Δf and ΔD shifts for Strep adsorption were larger and around −25.1 ± 0.1 Hz and 0.4 ± 0.1 × 10^−6^, respectively, for Strep 1. The corresponding values were −24.6 ± 1.1 Hz and 0.2 ± 0.3 × 10^−6^ for Strep 2. In addition, by applying the Sauerbrey equation (ref. [31]), it was determined that the surface coverage of Neu coverage per SLB area was lower than that of Strep (Figure 3F). Altogether, these data support that Step attachment to the biotinylated SLB occurs to a greater extent than that of Neu at pH 7.5.

Since Neu and Strep are both avidin-based proteins with similar sizes [42,43], the difference in adsorption uptake is likely related to variations in protein-SLB interactions and corresponding energetics. This finding led us to investigate how modulating the protein-SLB interaction strength might influence adsorption uptake, which led us to vary the solution pH. Indeed, since the pI of Neu is around 6.3, it would be more negatively charged at pH 7.5 than Strep, which has a pI around 7 and is hence nearly neutral at pH 7.5. Consequently, we reasoned that decreasing the solution pH to match the pI of Neu might improve its adsorption uptake.

#### 3.2.2. pH 6.3

**Figure 4:**
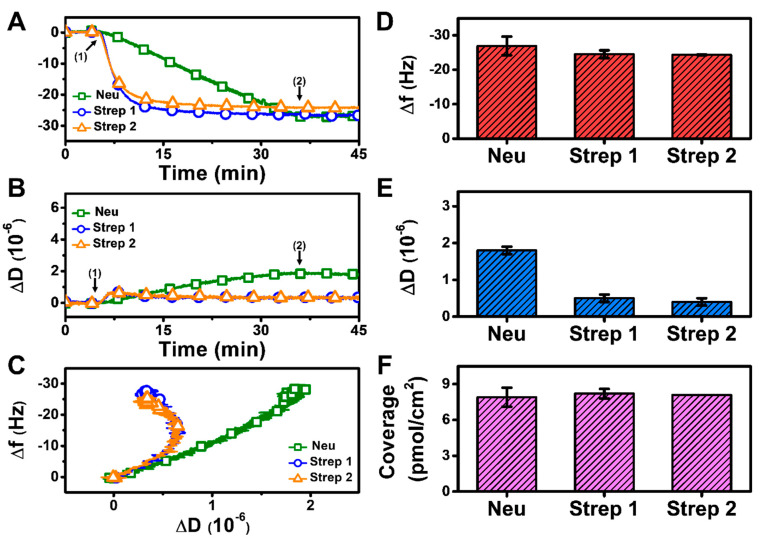
QCM-D measurement responses for Neu and Strep attachment to biotinylated SLB platform at pH 6.3. Time-resolved QCM-D (**A**) frequency (Δf) and (**B**) energy dissipation (ΔD) for protein addition. The initial baselines correspond to the shifts for a biotinylated SLB and protein was added starting at *t* = 5 min (arrow 1), followed by buffer washing from *t* = 35 min (arrow 2). (**C**) Time-independent curves of Δf vs. ΔD shifts corresponding to data in panels (**A**,**B**). Final (**D**) Δf and (**E**) ΔD shift values along with (**F**) maximum surface coverage of adsorbed protein molecules on the biotinylated SLB surface are reported as mean ± standard deviation from *n* = 3 experiments.

At pH 6.3, there was also monotonic protein adsorption for all three proteins while Strep adsorption again occurred more quickly and reached stable values, whereas Neu adsorption occurred more gradually and did not stabilize within 30 min (Figure 4A,B). Analysis of the f-D curves showed that the Strep adlayers are more rigidly attached to the biotinylated SLB platform than the Neu adlayer despite the increased Neu adsorption (Figure 4C). The final Δf and ΔD shifts for Neu adsorption were around −26.9 ± 2.7 Hz and 1.8 ± 0.1 × 10^−6^ (Figure 4D,E), respectively. For Strep 1, the final Δf and ΔD shifts were around −24.5 ± 1.1 Hz and 0.5 ± 0.1 × 10^−6^, respectively, and the corresponding values were −24.3 ± 0.1 Hz and 0.4 ± 0.1 × 10^−6^ for Strep 2. While the final Δf shifts and the corresponding surface coverage were similar for Neu and Strep, the larger ΔD shift for Neu adsorption indicates a more viscoelastic adlayer, possibly due to the attachment of Neu aggregates (Figure 4F). These data support that modulating solution pH can influence Neu uptake while there was negligible effect on Strep uptake.

#### 3.2.3. pH 8.7

**Figure 5:**
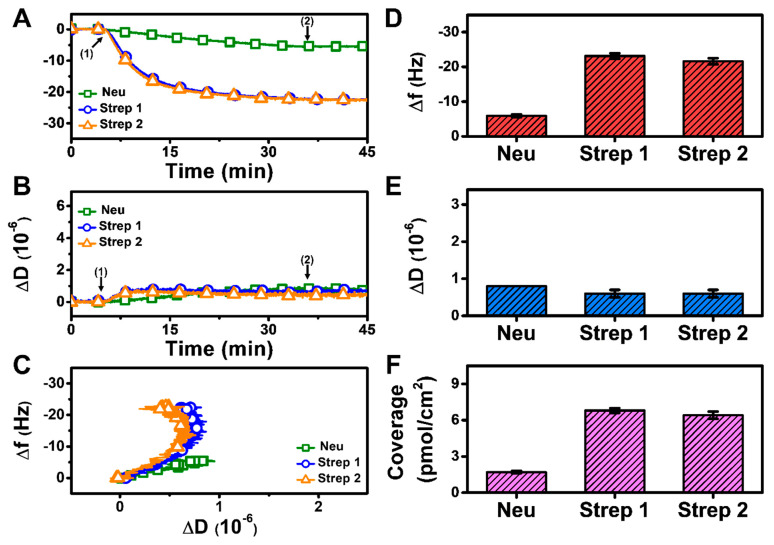
QCM-D measurement responses for Neu and Strep attachment to biotinylated SLB platform at pH 8.7. Time-resolved QCM-D (**A**) frequency (Δf) and (**B**) energy dissipation (ΔD) for protein addition. The initial baselines correspond to the shifts for a biotinylated SLB, and protein was added starting at *t* = 5 min (arrow 1), followed by buffer washing from *t* = 35 min (arrow 2). (**C**) Time-independent curves of Δf vs. ΔD shifts corresponding to data in panels (**A**,**B**). Final (**D**) Δf and (**E**) ΔD shift values along with (**F**) maximum surface coverage of adsorbed protein molecules on the biotinylated SLB surface are reported as mean ± standard deviation from *n* = 3 experiments.

To further test our hypothesis that modulating solution pH can influence Neu uptake based on the pI value, we also tested protein adsorption at pH 8.7 and observed appreciably less Neu uptake, whereas Strep adsorption was similar to the previous cases (Figure 5A,B). The corresponding analysis of the f-D curves also indicates a less rigid Neu layer (Figure 5C). The final Δf and ΔD shifts for Neu adsorption were only around −5.9 ± 0.4 Hz and 0.8 × 10^−6^, respectively (Figure 5D,E). By contrast, the final Δf and ΔD shifts for Strep 1 adsorption were around −23.1 ± 0.8 Hz and 0.6 ± 0.1 × 10^−6^, respectively, while the corresponding values were around −21.6 ± 0.9 Hz and 0.6 ± 0.1 × 10^−6^ for Strep 2. As expected, the surface coverage of Neu was less than that of Strep (Figure 5F). These results support that Neu uptake can be modulated by tuning the solution pH, which, in turn, influences protein–SLB interaction strength by adjusting the degree of electrostatic repulsion in the system. On the other hand, we observed a minimal effect of solution pH on Strep adsorption (mean Δf and ΔD shift deviation of only ~2 Hz and ~0.2 × 10^−6^, respectively, across the tested pH conditions).

We may also add a brief remark concerning how our Neu and Strep adsorption data compare to past QCM-D data reported in the literature. Previous results have shown that *nonspecific* Neu adsorption behavior onto inorganic surfaces can be variable and unpredictable, likely due to protein aggregation [44]. In our experiments that focused on *specific* binding of Neu and Strep to biotinylated SLBs, we used freshly prepared samples to minimize aggregation-related issues, while there were some differences in the trend in QCM-D responses observed in our study, as compared to past SLB studies. For example, we observed less Neu adsorption at pH 7.5 compared to other QCM-D data reported at similar pH conditions (i.e., pH 7.2) but with higher Neu concentration and/or biotin fraction in the SLB [45,46,47]. On the other hand, the QCM-D responses for Strep adsorption in our experiments are consistent with reported literature values in most cases [48,49,50,51]. These results also support the reliability of using Strep to functionalize SLB-based coatings via biotin coupling while Neu yields more variable results.

### 3.3. Kinetic Analysis

**Figure 6:**
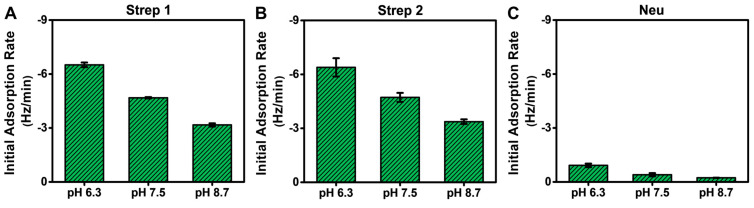
Initial adsorption rates for Neu and Strep protein binding to biotinylated SLBs in different pH conditions. The initial adsorption rates were calculated based on the time-dependent slope of the corresponding QCM-D Δf shift during the first 10 min of adsorption in all tested pH conditions for (**A**) Strep 1, (**B**) Strep 2, and (**C**) Neu. The rates are reported as the mean ± standard deviation from *n* = 3 experiments.

The QCM-D measurement analysis described above was mainly focused on adsorption uptake, and we observed that Neu adsorption occurred in a pH-dependent manner, which could be rationalized by taking into account its pI value. By contrast, Strep adsorption occurred in a pH-independent manner, which is intriguing because Strep adsorbed similarly even above its pI. With these points in mind, it is also noteworthy that the QCM-D results showed Step adsorption occurred more quickly than Neu adsorption in general, which led us to further analyze the initial adsorption kinetics based on the time-derivative of the QCM-D Δf shift response (Figure 6). The initial adsorption rate is influenced by the diffusion-limited rate of protein attachment and surface-induced protein denaturation [52,53]. Since all experimental parameters (flow rate, protein concentration, biotin fraction in SLB, etc.) were the same at each pH condition, changes in the initial adsorption rate could be attributed to pH-related changes in the protein-SLB interaction strength. 

While the total adsorption uptake was independent of solution pH for Strep proteins, interestingly, the initial adsorption rate was greater at lower pH, which is consistent with decreased electrostatic repulsion (Figure 6A,B). The corresponding values ranged from around −6.4 ± 0.5 Hz/min at pH 6.3 to −3.2 ± 0.1 Hz/min at pH 8.7, which reflects a roughly 2-fold variation. This trend supports that modulating solution pH also influences the rate of Strep adsorption by adjusting the protein–SLB interaction strength based on the pI, while the physical nature of Strep attachment to the biotinylated SLB occurs stably and similarly in all tested pH conditions. By contrast, the initial adsorption rate for Neu was much lower (less than −0.9 ± 0.1 Hz/min in all cases) and also occurred in a pH-dependent manner, with a >4-fold variation in rate values (Figure 6C). Hence, solution pH had a stronger effect on modulating Neu adsorption uptake, which further implies that the physical nature of Neu attachment to the biotinylated SLB is more variable depending on the environmental conditions and may also relate to the lower solution stability of Neu as well. 

**Figure 7:**
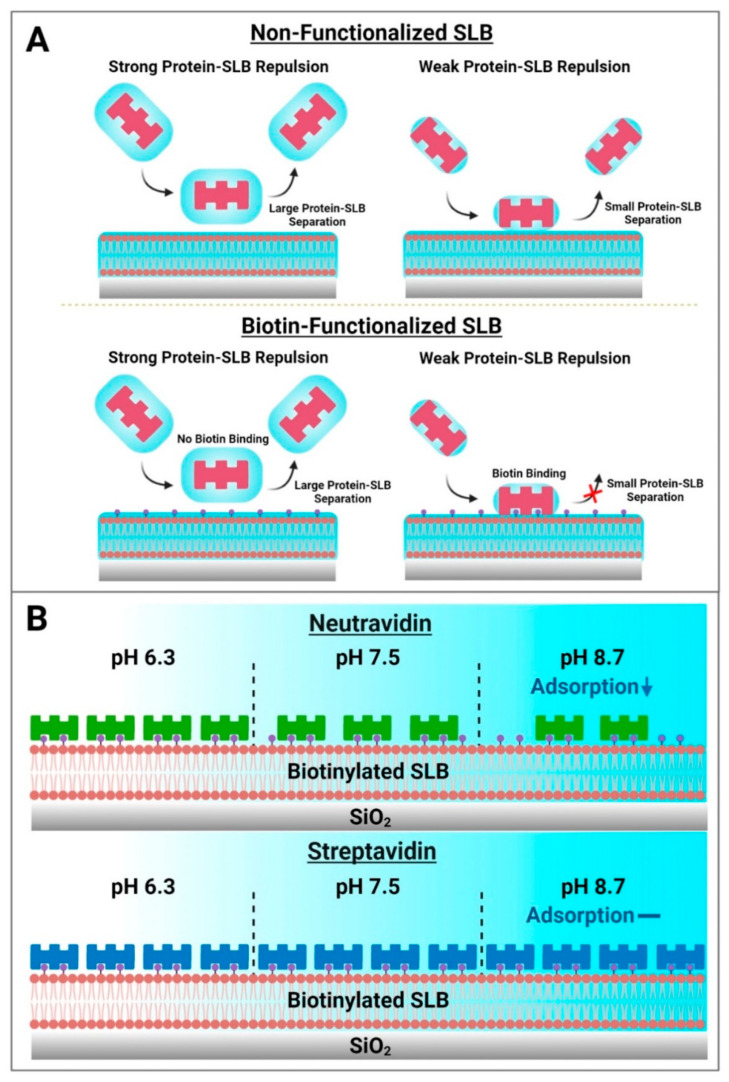
Summary of how biotin-binding proteins attach to biotinylated SLB surfaces and practical implications for biosensing applications. (**A**) Two-step model of how biotin-binding proteins come into contact with lipid bilayer surfaces. Electrostatic interactions modulate the magnitude of the equilibrium separation distance between contacting protein molecules and the SLB surface. In the absence of biotinylated lipid receptors, protein molecules may transiently contact a zwitterionic lipid bilayer but will not adsorb stably and the interaction is fully reversible independent of this separation distance. However, in the case of SLBs containing biotinylated lipid receptors, sufficiently close proximity of a contacting protein molecule to biotinylated lipid receptors (small separation distance) can result in strong binding, resulting in irreversible protein attachment. (**B**) Schematic illustration of Neu and Strep protein adsorption trends for SLB functionalization. Neu adsorption to biotinylated SLBs occurs in a pH-dependent manner, while Strep adsorption occurs in a pH-independent manner that can be useful for reliable surface functionalization.

Based on these results, a schematic summary of Neu and Strep adsorption onto biotinylated SLBs is presented in Figure 7. While protein attachment to the SLB platform in such cases is typically viewed in terms of binding to biotinylated lipid ligands alone, our findings paint a more nuanced picture whereby initial nonspecific protein adsorption to the SLB interface is modulated by the set of interfacial forces, including the double-layer electrostatic force, that govern interactions between protein molecules and the SLB interface, before protein binding to specific biotinylated lipids can occur (Figure 7A). From this perspective, the initial nonspecific adsorption process is a necessary step that precedes biotin coupling and requires the contacting protein molecules to come into sufficiently close proximity to the SLB interface, i.e., to the biotin-modified lipid headgroups slightly protruding from the lipid bilayer. In the Neu case, this initial adsorption step plays a more deterministic role in the overall attachment outcome and the amount of bound Neu protein was greater at pH 6.3, whereas there was appreciably less adsorbed protein at pH 8.7. On the other hand, the initial adsorption rate was high and relatively similar for Strep proteins, which exhibited more robust and stable attachment, in all tested pH conditions. It should also be emphasized that the initial nonspecific adsorption process itself is reversible and sensitive to the pH condition, whereas the resulting attachment is ultimately controlled by specific biotin coupling and is practically irreversible. In our experiments, this irreversibility was confirmed by observing no changes in the QCM-D responses even after exchanging pH conditions back to pH 7.5 in all cases. Collectively, the findings in this study support that Strep proteins are useful to form more stable adlayers on zwitterionic SLBs while the extent of Neu protein uptake can be modulated depending on solution pH (Figure 7B).

### 3.4. Biomacromolecular Detection

To investigate how the observed differences in Neu and Strep attachment translate into sensing applications, we further examined the detection of biomolecular interactions onto Neu- and Strep-functionalized SLB platforms, which were prepared at pH 6.3 (most favorable condition for Neu adsorption) and pH 7.5 (around the range of physiological pH conditions). In these experiments, we fabricated the Neu- and Strep-functionalized SLB platforms in the relevant pH condition and then performed a buffer washing step with pH 7.5 buffer, before adding 2 µM biotinylated bovine serum albumin (BSA) followed by 100 nM anti-BSA antibody to evaluate antigen-antibody detection [10].

**Figure 8:**
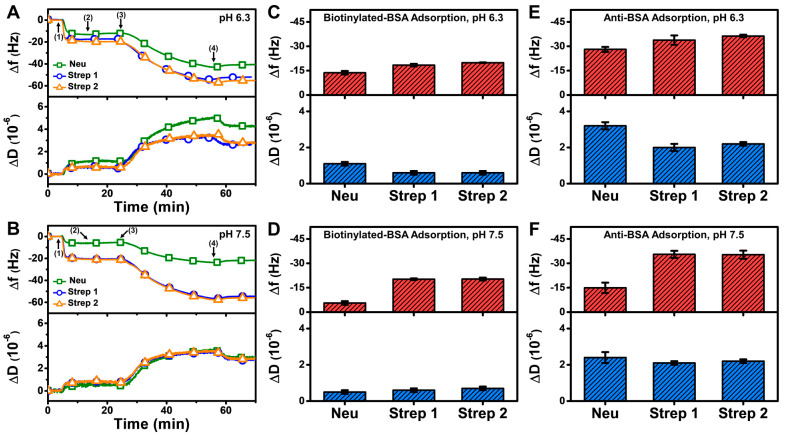
QCM-D measurement responses for biotinylated BSA and subsequent anti-BSA binding to Neu- and Strep-functionalized SLB platforms. Time-resolved QCM-D frequency (Δf, upper panel) and energy dissipation (ΔD, lower panel) for biotinylated BSA and anti-BSA adsorption onto Neu- and Strep-functionalized SLB platforms fabricated at (**A**) pH 6.3 and (**B**) pH 7.5. The initial baselines correspond to the shifts for a Neu- or Strep-functionalized SLB post-washing with pH 7.5 buffer and biotinylated BSA was added starting at *t* = 5 min (arrow 1), followed by washing with pH 7.5 buffer at *t* = 15 min (arrow 2), anti-BSA addition at *t* = 25 min (arrow 3), and washing with pH 7.5 buffer at *t* = 55 min (arrow 4). Final Δf (upper panel) and ΔD (lower panel) shifts for biotinylated BSA adsorption onto Neu- and Strep-functionalized SLB platforms that had been fabricated at (**C**) pH 6.3 and (**D**) pH 7.5, and corresponding data for subsequent anti-BSA adsorption in the (**E**) pH 6.3 and (**F**) pH 7.5 cases. All data are reported as mean ± standard deviation from *n* = 3 experiments.

The QCM-D results showed that the measurement responses for biotinylated BSA and anti-BSA detection on the Strep-functionalized SLB were moderately higher than on the Neu-functionalized SLB at pH 6.3 and appreciably higher at pH 7.5 (Figure 8A,B). The net Δf and ΔD shifts corresponding to biotinylated BSA adsorption onto the Neu-functionalized SLB at pH 6.3 were around −13.7 ± 1.1 Hz and 1.1 ± 0.1 × 10^−6^, respectively, and around −20 Hz and 0.7 × 10^−6^, respectively, on the Strep-functionalized SLB (Figure 8C). By contrast, the final Δf and ΔD shifts for biotinylated BSA adsorption onto the Neu-functionalized SLB at pH 7.5 were around −5.6 ± 1.1 Hz and 0.5 ± 0.1 × 10^−6^, respectively, while they were similar to the pH 6.3 results in the case of the Strep-functionalized SLB platform (Figure 8D). 

In addition, anti-BSA binding in the case of the Neu-functionalized SLB platform at pH 6.3 led to Δf shifts around −28.1 ± 1.5 Hz and ΔD shifts around 3.2 ± 0.2 × 10^−6^, while corresponding Δf shifts around −14.9 ± 3.2 Hz and ΔD shifts around 2.4 ± 0.3 × 10^−6^ occurred at pH 7.5 (Figure 8E,F). On the other hand, the measurement responses were appreciably larger for Strep-functionalized SLB platforms, and the corresponding Δf and ΔD shifts were around −36 Hz and 2 × 10^−6^, respectively. Taken together, these results demonstrate that Strep proteins have superior and more reliable performance for antigen-antibody detection involving SLB platforms and are hence preferable over Neu for such biosensing applications.

Regarding the applicability of these scientific results to various SLB platforms, it should be noted that the results were obtained using a zwitterionic SLB, which is widely known to have antifouling properties [19] and is hence routinely used. Depending on the sensing application or other biophysical objective, it is possible to change the lipid composition, which might affect the protein attachment specifics. For example, the initial nonspecific adsorption stage might be either promoted in cases where the SLB contains lipids with charged headgroups that allow for greater electrostatic interactions with oppositely charged protein domains, or reduced in cases where the SLB contains saturated lipids that have a higher packing order and thus restrict lateral movement of the SLB for protein binding [54]. Future studies are warranted to further investigate the effects of the SLB lipid composition (as well as other operating parameters such as temperature, metal ions, and interfering proteins/small biomolecules in the buffer) on protein binding, while expanded application use will likely involve defining standardized protocols for reliable protein attachment. Moreover, other types of SLB that are not directly adhered to the substrate for certain applications (e.g., tethered SLBs with polymer cushions or ionic reservoirs [55,56]) can be explored to better understand protein binding efficiency and functionalization suitability thereof for targeted applications.

## 4. Conclusions

In this study, we investigated the effects of solution pH on Neu and Strep protein binding to biotinylated SLBs using the QCM-D technique. Our findings support that Neu adsorption occurs relatively slowly and in a pH-dependent manner, both in terms of the initial adsorption rate and total uptake. In marked contrast, Strep adsorption occurred more quickly and its uptake amount did not depend on the solution pH, i.e., there was a similar amount of bound Strep at all tested pH conditions. Of note, the initial adsorption rate of Strep did depend on the solution pH although the variation in rate magnitude was relatively small. As such, the initial rate of both Neu and Strep both occurred in a pH-dependent manner, which can be rationalized by taking into account the pI of each protein and the relevant interfacial forces in the system, especially the double-layer electrostatic force. While it is often assumed that Neu and Strep have equivalent and interchangeable functionalities due to similar biotin-binding abilities, our findings demonstrate that Neu and Strep attachment to biotinylated SLB interfaces is quite different in terms of attachment kinetics, adlayer properties, and pH sensitivity. The results support that Strep is a more reliable option for SLB functionalization in biosensing application contexts on account of quick attachment and robust adlayer properties and such insights can be broadly useful for surface functionalization involving the attachment of biotin-binding proteins at biotinylated sensing interfaces.

## Data Availability

The data presented in this study are available upon reasonable request from the corresponding authors.
